# Progress in Fevers

**Published:** 1901-04-13

**Authors:** 


### PROGRESS IN FEVERS.

(*Continued from page 9.*)

**Plague.** — Cantlie[20] describes three main types—bubonic, pneumonic, septicæmic. Buboes appear in at least 70 per cent. Infection is conveyed by rats, parasites, and man. Malaise may accompany the incubation period of three to five days. Onset is sudden, with rigors, headache, pain, prostration, fever, and mental disturbance. Buboes appear the third day. Furred tongue, vomiting, constipation, followed by diarrhœa and enlarged spleen are present; breathing is hurried, but lung complications are rare. Apathy, sleeplessness, delirium, mania, muscular incoordination and twitching are common. "Thickness of speech, due evidently to lingual paresis, is almost a diagnostic feature." Apyrexia is the rule in pestis ambulens and fulminant or septicæmic plague. Dyspnœa, cough, fever, and prostration mark the pneumonic form, evanescent patches of lobular pneumonia occur, the sputum swarms with bacilli. Death is common on the third or fourth day. The septicæmic form occurs with sudden extreme prostration, frequently apyrexia, delirium, and coma. Death commonly occurs the second or third day. *Post-mortem*: Polyadenitis, enlarged spleen, generally congested viscera, serous and mucous hæmorrhages are found. In the pneumonic form patches of consolidation occur but no adenitis. Robertson[21] describes as special diagnostic features, anxious face, bloodshot eyes, thick, hesitating, broken speech, and tongue with creamy (later brown) fur, and red tip and edges (Simpson). Colvin[22] discusses the diagnosis between plague and typhoid. Buboes and Widal's reaction are distinctive. Sudden onset, irregular fever, dyspnœa out of all proportion to physical lung signs, early nervous symptoms with marked restlessness favour plague. Tenderness in the cæcal region suggests examination of the right groin. Savage and Fitzgerald[23] report a case with painless enlarged glands and "carbuncles" on the shins. Calmette[24] regards broncho-pneumonia as constantly present after three to four days. Hewlett[25] says rapid changes in the tongue are typical (Clemow). D. Rees[26] describes the bacillus as a short fat rod with rounded ends, often in pairs, rapidly killed by sunlight and disinfectants. Pakes[27] emphasises the need for care in experimenting with it. Fraser[28] lays stress on the mild ambulent case as a cause of infection. In India improved housing and sanitation, letting in air and sunlight are needed: chemical disinfection in the present style of native dwellings seems hopeless. Total evacuation of villages stricken and observation of ports alone do good.

Lustig and Galeotti[29] employ for prophylaxis the nucleo-proteid extracted from the bodies of plague bacilli; the serum of animals so inoculated is used as a curative serum. Calmette's[30] "vaccine" and serum are similar, but he uses the entire bacillus. The curative dose is 20 c.c. injected into a superficial vein, repeated in twenty-four hours if necessary. 10 c.c. as a prophylactic may be injected under the skin of the abdomen. B. Stewart[31] describes Haffkine's immunising fluid as a vaccine, consisting of dead microbes with their culture medium. Yersin's serum only confers immunity for fifteen days. M. T. Christie[32] gives details of the antiseptic method of using Haffkine's prophylactic in Calcutta. Delepine[33] has produced different forms of plague by varying the seat of inoculation.

[20] Brit. Med. Jour., Oct. 27. [21] Quar. Med. Jour. Nov. [22] Lancet, Oct. 27. [23] Brit. Med. Jour., Oct. 27. [24] Lancet, Nov. 10. [25] Treatment, Nov. [26] Brit. Med. Jour., Oct. 27. [27] Ibid. [28] Brit. Med. Jour., Nov. 17. [29] Brit. Med. Jour., Jan. 26. [30] Lancet, Nov. 10 and 17. [31] Brit. Med. Jour. Oct. 27. [32] Ibid. [33] Ibid.

